# Barriers to cancer symptom presentation among people from low socioeconomic groups: a qualitative study

**DOI:** 10.1186/s12889-016-3733-2

**Published:** 2016-10-05

**Authors:** Grace McCutchan, Fiona Wood, Stephanie Smits, Adrian Edwards, Kate Brain

**Affiliations:** Division of Population Medicine, School of Medicine, Cardiff University, Cardiff, UK

**Keywords:** Cancer, Cancer awareness, Cancer beliefs, Symptom presentation, Symptom assessment, Socioeconomic status, Help-seeking behaviour, Patient interval

## Abstract

**Background:**

Socioeconomic inequalities in cancer survival can in part be explained by long patient intervals among people from deprived groups; however, the reasons for this are unclear. This qualitative study explores the actual and anticipated barriers to cancer symptom presentation in the context of socioeconomic deprivation.

**Methods:**

Thirty participants were recruited through the International Cancer Benchmarking Partnership Welsh database (*n* = 20), snowball sampling (*n* = 8) and community partners (*n* = 2). Semi-structured qualitative interviews were conducted with symptomatic and asymptomatic adults over the age of 50 years, who were identified as being from a low socioeconomic group based on multiple individual and group level indicators. Transcripts were analysed using a Framework approach based on the COM-B model (Capability, Opportunity, Motivation-Behaviour).

**Results:**

There was evidence of poor awareness of non-specific cancer symptoms (Capability), fearful and fatalistic beliefs about cancer (Motivation), and various barriers to accessing an appointment with the family physician (Opportunity) and full disclosure of symptoms (Capability). These in combination were associated with a lengthened patient interval among participants. Social networks (Opportunity) were influential on the formation of knowledge and beliefs about cancer. Participants’ behavioural and normative beliefs were usually formed and reinforced by people they knew with cancer, and such beliefs were considered to lengthen the patient interval. Discussing symptoms with a family member or friend before a visit to the family physician was the norm, and could act as a barrier or facilitator depending on the quality of advice given (Opportunity). Economic hardship meant fulfilling basic day-to-day needs such as finding money for food were prioritised over medical help seeking (Opportunity).

**Conclusions:**

The complex interaction between individual characteristics and socio-environmental factors is important for understanding cancer symptom presentation behaviour, especially in the context of socioeconomic deprivation. Interventions targeted at deprived communities should take into account the wider social influences on symptom presentation behaviour.

## Background

Socioeconomic inequalities in cancer outcomes exist, where the chances of surviving cancer decrease with increasing deprivation [1–4]. This is likely to reflect a higher incidence of cancers with poor prognosis, such as lung cancer [2, 5, 6] and later stage disease at diagnosis in low socioeconomic groups [2, 7, 8], as a consequence of prolonged cancer symptom presentation [9]. For example, in the UK 11 % more cases of lung cancer occur in the most income deprived group compared to the least income deprived group, and the relative difference for 1 year survival between the highest and lowest socioeconomic groups is 2.6 % [2]. It has been estimated that the socioeconomic inequalities in cancer survival account for more than 7000 lives in England annually [1].

Early detection of cancer can improve survival outcomes, and in part relies on prompt cancer symptom presentation [2]. The patient interval - defined as the time between symptom discovery and the initial visit to a health care professional - accounts for the longest period of time between symptom detection and start of treatment [10–13]. Although the patient interval has been found to lengthen with increasing socioeconomic deprivation [9], the reasons why people from a low socioeconomic group attenuate the decision to present with cancer symptoms are not fully understood. Insight into the factors underlying a long patient interval is essential for the development of targeted interventions to promote timely cancer symptom presentation among low socioeconomic groups and facilitate earlier diagnosis of cancer.

The (UK) National Awareness and Early Diagnosis Initiative (NAEDI) framework was developed to test hypotheses relating to premature deaths from cancer [14]. It is a descriptive framework which suggests that poor knowledge, negative beliefs and barriers to help-seeking result in a long patient interval [14]. Recently the NAEDI framework was updated to include socioeconomic characteristics as risk factors for a longer patient interval [14]. Empirical evidence to support the NAEDI framework has been reported, including lower cancer symptom knowledge [15–19], lower suspicion of cancer when experiencing symptoms [20], a higher prevalence of fearful and fatalistic beliefs about cancer [21–23] and emotional barriers such as fear of diagnosis or embarrassment around disclosure of symptoms [16, 18, 24] among low socioeconomic groups. These factors in combination are likely produce a long patient interval [25]. However, the descriptive nature of the NAEDI framework does not provide insight into how socioeconomic factors might influence knowledge, beliefs, barriers and symptom presentation. Evidence regarding the influences on symptom presentation behaviour has mainly been restricted to studies using quantitative methods, involving samples with limited socioeconomic variation and often relying on a sole indicator of deprivation. In addition, studies have typically focused on individual barriers rather than the wider socio-environmental determinants of behaviour [15, 16, 18, 21, 22, 24].

A more detailed understanding of how both individual and socio-environmental factors might lengthen the patient interval is required. The COM-B model offers a potentially useful insight into how the decision to present with a potential symptom of cancer might be influenced through the constructs of ‘Capability’, ‘Opportunity’ and ‘Motivation’ [26]. Where many other theories neglect the wider social influences on behaviour, the COM-B model takes these and other individual level constructs into account, and was selected to aid analysis and interpretation of the data. According to the COM-B model [26], in order for behaviour to occur, an individual must have the ‘Capability’ (physical or psychological capacity of a person to perform behaviour) as well as the ‘Opportunity’ (physical opportunities created by the physical environment or social opportunities created by the cultural environment). In addition, ‘Motivation’ to engage in the target behaviour must outweigh motivation to engage in competing behaviours [26]. ‘Motivation’ may be automatic (Type 1 innate, unconscious processes *e.g.* habitual or emotional responses) or reflective (Type 2 deliberative, slower processes *e.g.* conscious decision making) [26].

To our knowledge, no study to date has sought to understand the actual and anticipated barriers to cancer symptom presentation from an in-depth qualitative perspective with participants from a low socioeconomic group. We aimed to explore cancer symptom knowledge, beliefs about cancer, the wider social determinants and barriers to actual and anticipated presentation of cancer symptoms in a sample of participants from a low socioeconomic group, identified using multiple socioeconomic group indicators. Symptomatic and asymptomatic participants were included to understand how these factors might affect actual or anticipated cancer symptom presentation behaviour.

## Methods

### Participants

Participants were initially sampled from the International Cancer Benchmarking Partnership (ICBP) Welsh database of participants who previously took part in a population-based cancer awareness telephone survey [15, 27]. Participants were sampled purposively, stratified by Welsh Index of Multiple Deprivation (WIMD) score and educational attainment to ensure that those from a low socioeconomic group were invited to take part in the study. Individuals from a low socioeconomic group were defined as those residing in the most deprived quartile classified by WIMD score, and with the lowest educational attainment (‘finished school before age 15’ or ‘no qualifications/left school at age 16’). Due to low response rates using ICBP database recruitment, snowball sampling was used to recruit additional participants opportunistically through previously recruited participants, and through ‘Communities First’ health leads who are contracted by the Welsh Government to support people living in the most disadvantaged communities in Wales. Additional individual and area level socioeconomic group indicators were collected at interview, including main source of income, car and home ownership. All participants were over the age of 50 years, in order to reflect the age at which cancer becomes more common [27]. Participants who reported a previous cancer symptom episode and those who disclosed a past diagnosis of cancer during the interview were included, along with those who reported no prior cancer symptom experience during the interview. This was to gain insight into actual and anticipated barriers to cancer symptom presentation among participants with a range of cancer symptom experience.

### Procedure

Potential participants were initially contacted by telephone and invited to take part in the study. Those who expressed an interest were posted information about the study, and a follow-up phone call was made a week later to arrange a time and date for interview. Ethical approval was received from the Cardiff University School of Medicine Research Ethics Committee (reference 14/01). Participants were offered £10 to thank them for their time. Face-to-face (*n* = 26) and telephone (*n* = 4) semi-structured qualitative interviews were carried out between June 2014 and March 2015 until data saturation was achieved (no new themes emerged from interview analysis). Face-to-face interviews took place in participants’ homes or a place of their choosing following written consent. Verbal consent and written postal consent were obtained prior to telephone interviews. The interview topic guide was informed by a systematic review conducted by the authors [25] and refined following initial interviews. Topics under discussion were: cancer knowledge, beliefs about cancer, experience of cancer, actual or anticipated symptom presentation, barriers and facilitators to symptom presentation, current cancer campaign awareness, suggestions for intervention and symptom disclosure within the community. Participants who disclosed a previous diagnosis of cancer or symptom episode which they considered to be indicative of cancer were asked about their presentation behaviour (if and when they had consulted a doctor about the symptom). Those who reported no symptoms were asked to consider if or when they would seek medical help for a potential symptom of cancer. All study materials, including the interview topic guide, were developed with a member of the public volunteering for a cancer charity. The volunteer was asked to provide feedback around readability of study recruitment materials and comprehension of interview questions. Interviews were conducted by GM, a health psychology PhD student trained in the design and conduct of qualitative interviews. Interviews were audio-recorded and were between 45 min and 2.5 h (mean 72 min) in duration.

### Analysis

Verbatim transcripts were analysed using a framework approach [28] based on the COM-B model [26]. Themes were identified from the transcripts and clustered under each of the COM-B model constructs (Capability, Opportunity, Motivation-Behaviour) [26, 28]. Four transcripts (13 %) were independently double coded, with discrepancies resolved through discussion. Data were managed using NVivo 10 [29].

## Results

Thirty participants were interviewed (13 men and 17 women), with a mean age 66 years (range 52 to 88 years). All participants resided in the most deprived areas in Wales and were representative of a low socioeconomic group based on other indicators (see Table 1). Participants reported a range of symptom experience and actual/anticipated time to symptom presentation. Five participants reported a previous diagnosis of cancer, 16 participants reported a previous symptom of cancer, and nine reported no previous symptoms of cancer.

**Table 1:** Sample characteristics

Characteristics	Participants
Recruitment source	ICBP Welsh database (*n* = 20; 26 % response rate calculated as a proportion of those eligible for the study after verbal contact was made) Snowball sampling (*n* = 8) ‘Communities First’ Partners (*n* = 2)
Gender	Female (*n* = 17) Male (*n* = 13)
Age	50–60 years (*n* = 10) 61–70 years (*n* = 13) 71–80 years (*n* = 5) 81–90 years (*n* = 2)
Symptom experience	Previous diagnosis of cancer (*n* = 5) Reported cancer symptoms (*n* = 16) No cancer symptom experience (*n* = 9)
Educational attainment	Finished school before age 15 (*n* = 15) No qualifications or left school at age 16 (*n* = 15)
Main source of household income	Wages or salary (*n* = 3) Pension (*n* = 18) Benefits (*n* = 8) Other (*n* = 1)
Home ownership	Owns home (*n* = 6)^a^ Privately rented housing (*n* = 11) Housing association or sheltered housing (*n* = 7) Council owned property (*n* = 6)
Car ownership	Owns car (*n* = 9) Does not own car (*n* = 21)

^a^These participants had inherited the family home, where they had lived their entire lives

### Capability

#### Cancer knowledge

Participants attributed non-specific symptoms such as weight loss or fatigue to existing co-morbid illnesses that were highly prevalent in this group, such as diabetes, and perceived no urgency to seek medical help for these symptoms. Four participants with a previous diagnosis of cancer were unaware that their early symptoms were warning signs for cancer. Instead, symptoms were attributed to benign causes such as the menopause, which led to a patient interval of up to 11 months. Two participants reported receiving a cancer diagnosis whilst under investigation for another health complaint which they perceived as unrelated. Four participants expressed anxiety around the belief that some cancers were symptomless. Twenty participants discussed potential causes for cancer that were beyond their control and therefore expressed a reluctance to change ‘risky’ behaviours due to a perceived lack of benefit. Many of these participants thought that “we’re all born with cancer in us” *(Female, age 62, previous diagnosis of cancer, diagnosed incidentally through secondary care*) where trauma such as a hit, bump, psychological stress or chemicals used in food was required to “trigger [cancer] off” (*Female, age 66, cough and reflux symptoms reported, did not seek medical help*) or that luck could determine who was ultimately diagnosed with cancer:

“The thing is how do you know when you’ve got [cancer] anyway? You know what I mean? You don’t know really, until it reacts with you, know what I mean?” (*Male, age 80, cough symptom reported, did not seek medical help*)

“[Cancer is] in everything we eat…using different fertilisers to make [food] grow to keep the flies and that away…You read all this in the paper, eat this, eat that, it’s healthy for you, but it’s the sales patter to sell it I’m sure, because it’s not doing anyone any good…I say “eat what you like, eat it, if you like it eat it”…[cancer is] in what we eat, but you’ve got to eat, it’s as simple as that.” (M*ale, age 72, change in bladder habit reported, did not seek medical help*)

“I never even heard of that there’s something wrong with your prostate, I didn’t even know what the prostate was…I was thinking about going to the toilet all the time?” (M*ale, age 75, previous diagnosis of cancer, sought help after 6 months*)

#### Communication with Healthcare Professionals

Eleven participants perceived themselves as having the capacity to effectively communicate symptoms to a Healthcare Professional (HCP), sometimes using prompts such as lists to facilitate discussion. In addition, they felt confident about actively participating in a discussion with a HCP around healthcare options. For these participants, effective communication was perceived as important for access to optimal healthcare provision. Other participants preferred to take a more passive role in their healthcare, expressing frustration when invited to participate in a discussion with a HCP about potential diagnosis and management of symptom(s). A passive approach to healthcare was perceived as the norm within the community. Some participants described a lack confidence when communicating symptoms or were “in awe of their GP” (*Female, age 52, no symptoms reported, would seek medical help quickly if appraised symptom as cancer*). They also struggled to communicate concerns or ask questions, which could reflect a power imbalance in the doctor/patient relationship and low literacy in this group, and has the potential to lengthen the patient interval:

“[The doctors] say to you “what do you think?” and as I say to them, “I’m not the doctor how do I know?” If it comes to diagnosing yourself why bother going to them? You know, what’s the point?” (*Female, age 66, cough and reflux symptoms reported, did not seek medical help*)

“I’m the type of person, I question something, [my husband] will accept it more than I will, he’ll say “oh well I’ve been told, listen now they’ve told me and that’s the end of it” [I say] “no it’s not the end of it, you disagree with it or you don’t believe it, question it again.” *(Female, age 57, no symptoms reported, would seek medical help quickly for cancer symptom*)

“You can write down what you want to discuss with the doctor while you are waiting…because sometimes if you don’t write them down, and you go there you forget.” (*Female, age 71, no symptoms reported, would seek medical help quickly for lump and bleeding symptoms*)

### Motivation

#### Fearful and fatalistic beliefs about cancer

When participants were asked for their initial reactions to cancer as a disease, all participants’ initial, ‘automatic’ (Type 1) reactions to the word cancer were fearful and highly emotive, where participants described cancer as “evil” or “terrible”. For four participants reporting lump symptoms, fear prompted immediate actual symptom presentation. For three symptomatic participants, fear was reported to lengthen the patient interval, where there was a need to come to terms with a potential diagnosis of cancer prior to seeking medical help. Eleven participants expressed fatalistic beliefs about cancer such as the belief that death is inevitable after receiving a diagnosis, and that treatments can only prolong life rather than cure cancer. Such beliefs were often supported with anecdotal accounts of friends and family who suffered and died from cancer. Participants perceived fear to be the biggest barrier to cancer symptom presentation in the community, especially when combined with fatalistic beliefs about cancer:

“It took me a long time to go [to the doctor] I know that, I was terrified…I was terrified of the answers…what I don’t know I can’t worry about can I?” (*Female, age 88, previous diagnosis of cancer, sought medical help after a few months- unable to recall exact time*)

“All I know is that once you get it, that’s your lot, as far as I know there is no cure …it’s a dirty disease isn’t it? That’s the description of cancer, it’s a dirty disease…you start thinking the worst and to be honest with you the worst is cancer! No one thinks of heart attacks, or fits, or strokes, the first thing is cancer. Phone the funeral director I’ve got cancer!” (*Male, age 80, cough symptom reported, did not seek medical help*)

“[People are] afraid I think it is to find out the truth. They know there’s something wrong, they’re just afraid to actually hear the doctor come out and say the word “cancer”…They’re afraid to go to the doctor’s in case they actually say “yes, you have got cancer” and a lot of people are afraid to hear that word you know…so a lot of them will just sort of put it off until they’re so ill they’ve got to go.” (F*emale, age 52, no symptoms reported, would seek medical help quickly if appraised as cancer*)

#### Beliefs about treatments and early diagnosis

Most participants were fearful of treatments for cancer and seven believed the treatments to be worse than the cancer itself or thought surgical procedures allowing air to come into contact with the tumour could cause the cancer to spread. Three participants would refuse treatment if diagnosed with cancer. As each interview progressed, twenty participants discussed the benefits of early diagnosis, where seeking medical help quickly was considered important to enable access to less invasive treatments and potential cures for cancer. However, curative treatment was only considered possible for certain “good” cancers such as breast and prostate if caught in the early stages. Positive beliefs were disclosed even after expressing previous fearful beliefs about cancer. These contradictory beliefs are likely to reflect a deep-seated and potentially irreversible fear of cancer. Participants who held the belief that certain cancers were asymptomatic in the early stages, in combination with an understanding of importance of early diagnosis expressed anxiety. This was due to the perception that early diagnosis of cancer was beyond their control because some cancers were only symptomatic in the later stages, where a cure is less likely. This could reflect the high incidence of cancers with worse outcomes within their community, such as lung or pancreatic cancer, and which are harder to diagnose in the early stages:

“It all depends on what cancer it is, if it was breast cancer I think you’ve got a good chance, if you got it early enough, bowel cancer is a good cancer if you’ve got to have cancer.” (F*emale, age 69, various gastric symptoms reported, sought medical help after 1 month*)

“My sister she had radio, whatever you call it....on her throat and that when she had the throat cancer and when we went to see her, she was burned inside and outside, and it makes them ill and sick and whatever. Well a lot of people with cancer would rather die from the cancer than go through the treatment.” (*Female, age 52, no symptoms reported, would seek medical help quickly if appraised as cancer*)

### Opportunity

#### Facilitators to a short patient interval

Nine participants reported a good relationship with their family physician, where they felt their doctor was interested in them as a patient, listened and were easy to talk to. These participants felt confident in presenting to their doctor with symptoms to discuss concerns and perceived this as a key facilitator to symptom presentation. Ten participants benefited from family members or friends who helped to book an appointment with the family physician, arrange transportation to an appointment, or communicate symptom concerns during an appointment. A few participants knew that primary care had emergency appointments, which could be used to discuss worrying symptoms:

“I can talk to [my wife] better than if I go to the doctor and then [my wife] could take me down to the doctor…she’d do the talking and all this and that cos I clams up, I’m not a very good talker, she’s a better talker than me.” (*Male, age 67, cough symptom reported, did not seek medical help*)

“If it was me and I had any symptoms like that I would go straight and I wouldn’t hesitate in going to my GP. As I say I’ve got a good GP here.” (*Female, age 60, no symptoms reported, would seek medical help immediately for a lump symptom)*

#### Barriers to symptom presentation

Lack of continuity of care was described as a barrier to symptom presentation. Participants weighed up the problems with seeing a different doctor on every visit, where they were required to explain the same problem over again, against waiting two to three weeks for an appointment with their preferred family physician. For thirteen participants, the practicalities of getting to an appointment were barriers to symptom presentation because of a lack of transportation, work commitments and physical disabilities. Loss of earnings due to attending an appointment during work hours lengthened the patient interval with symptoms that were perceived as non-urgent. Unpredictable shift patterns were likely to make planning and scheduling an appointment difficult. Time limited appointments, and ‘one appointment, one problem’ policies prohibited the disclosure of more than one symptom. This was frustrating for participants, especially when they took time off work or waited a few weeks for an appointment. Consequently, participants perceived there to be little point in going to their family physician where time limited appointments restricted discussion of all health complaints, which has the potential to stop a future presentation or lengthen the patient interval:

“They don’t pay you to go to the doctor, you’ve got to clock in and clock out. I said "no, I can’t afford to lose time off work” and I don’t drive for another thing, so I said “where it would take 10 min to get down to you, I’ve got to wait for a bus, get down on the bus, and then go back to work which would take me an hour.” (*Female, age 57, lump symptom reported, sought medical help after 2 months*)

“The last three times I’ve been now I’ve seen three different doctors…I suppose if I was prepared to wait, I could see the same one” (*Male, age 81, previous diagnosis of cancer, diagnosed incidentally through secondary care*)

“I went in there with a complaint and he’s seven minutes. I said I got something else and he said “you’ve already come for the one complaint, you’ll have to make another appointment to see me again”.” (*Male, age 80, cough symptom reported, did not seek medical help*)

#### Experience of cancer

Participants’ experiences of cancer from others in the community acted as Opportunity to gain and reinforce knowledge and beliefs about cancer. To support beliefs about cancer or demonstrate knowledge for the symptoms and causes of cancer, participants almost exclusively drew upon anecdotal accounts of people they knew with cancer in the community, which were generally negative. Participants recalled many family members and/or friends in the community who suffered or died from cancer, which is likely to contribute to the formation of fearful and fatalistic beliefs about cancer. Four participants knew one or two people in the community who survived cancer, but who had kept their diagnosis a secret due to perceived stigma. In addition, the only cancer related media coverage that most participants could recall was about celebrities who died from cancer. It is unlikely that this was the only media coverage about cancer that participants had seen but importantly, these were the most memorable and are likely to reinforce negative beliefs:

“It’s a waste of time, you’ll never cure [cancer], I’ve had seventeen in the family die of it.” (*Male, age 56, no symptoms reported, would not seek medical help for symptoms*)

“[My father] died in agony. I was there and he was, I’ll never forget it on [date] I watched him die in agony, like I watched my wife [die from cancer], through incompetence you know…” (*Male, age 72, change in bladder habit reported, sought medical help within 1 month*)

#### Lay advice in healthcare

Seeking advice for symptoms from family members or friends before visiting doctors was the norm, whether to seek reassurance or decide whether an appointment with the family physician was required. This is likely to reflect the various reported barriers such as the practicalities of getting to the doctor, to ensure an appointment is necessary. Participants would discuss symptoms with those close to them or those perceived within the local community to be ‘knowledgeable’, such as the local hospital transport driver or someone with cancer. Fifteen participants described people within the community asking them for advice on symptoms, or noticing symptoms in others. Depending on the quality of advice given, symptom disclosure could act as a barrier or facilitator to symptom presentation:

“Well the first person I’d talk about [a symptom] to is my mate because she’s had a couple of scares and fortunately thank God she hasn’t got cancer and then the next person I would go and see is my doctor.” (*Female, age 70, unexplained weight loss, sought medical help after 2 months*)

“[My friend] had been complaining that she wasn’t well before Christmas but she’s so stubborn that she wouldn’t go to the doctors and we kept on saying to her “go, it’s not normal to lose this amount of weight in such a short time”, and she said “oh I’ll go now” and she did go now mind.” (*Female, age 57, lump symptom reported, sought medical help after 2 months*)

#### Social environment

For many participants health was not perceived as a priority. Instead, day-to-day problems took precedence such as finding money for food and heating the house. There was an overall sense of suspicion and lack of trust towards the government, where some participants believed the government to be withholding the cure for cancer, or were ‘playing God’ through ‘postcode lotteries’ where access to certain cancer treatments was determined by area of residence. Some participants discussed feeling victimised or forgotten by the government. Competing priorities, a lack of resources available for a healthier lifestyle, and perceived lack of control over daily life are likely to impact on help-seeking behaviours:

“They’re on about we’ve got cures for this, cures for that, I think it’s just a big money making thing to be honest, I think that it’s a case of they got it and we ain’t sharing it because there’s too much money going in....” (*Male, age 56, no symptoms reported, would not seek medical help for cancer symptoms*)

“I get angry because they cut everybody else’s money back except the politicians and they get money and some of these have got three and four houses, cars, I’m thinking alright, why do you need all those houses?” (*Female, age 52, no symptoms reported, would seek medical help quickly if appraised symptom as cancer*)

“Your health goes because myself right, I need £10 for the gas and I’ve got £20 in my purse to last me the week, but it’s gonna cost me £15 to get fresh veg, meat and fruit. Then I would leave the fruit aside and the veg, to make sure that I’ve got my gas to keep warm.” (*Female, age 57, no symptoms reported, would seek medical help quickly for symptoms*)

## Discussion

This qualitative study provides rich insight into cancer knowledge, beliefs about cancer and the barriers/facilitators to cancer symptom presentation to understand how these might lengthen the patient interval among people from low socioeconomic groups. Some participants had experienced cancer or potential symptoms of cancer and some participants had not and were considering symptom presentation. Knowledge of cancer and fearful and fatalistic beliefs, where participants associated cancer with inevitable death, were usually formed and reinforced by witnessing family and friends suffer and often die from cancer. Poor knowledge and fearful and fatalistic beliefs about cancer were associated with longer actual or anticipated patient intervals. In contrast, those who held positive beliefs about the benefits of early diagnosis could quickly overcome any reported practical and service barriers for ‘red flag’ symptoms such as a lump [30], following accurate symptom appraisal. Most participants who reported a previous lump symptom sought medical help quickly and those with no symptom experience anticipated seeking medical help quickly for a lump symptom. However, for non-specific symptoms that were usually attributed to symptoms of other co-morbid illnesses, competing priorities such as work commitments were often more influential on the individual’s decision about whether to seek help. This resulted in a long patient interval. Disclosure of symptoms to family or friends could further lengthen the patient interval or prompt early symptom presentation, depending on the nature of advice received.

Our findings confirm those of previous studies conducted in high income countries with wider participant samples of various socioeconomic groups and a range of tumour sites. Poor knowledge of non-specific cancer symptoms [15, 16], fearful and fatalistic beliefs about cancer [21, 22] and emotional barriers to cancer symptom presentation [16, 18, 24] were more prevalent in low socioeconomic groups. Through in-depth qualitative methods, we provided insight and possible explanations for these findings by exploring the wider social context (‘Opportunity’) that is specific to low socioeconomic groups, and how this influenced cancer symptom presentation. General fatalistic attitudes were common, where individuals believed themselves to have little control over their fate. Consequently, there was a reluctance to change risky health behaviours, and the potential for individuals to deny or ignore health problems. For some, this extended to cancer-specific fatalism in which the patient interval was lengthened because cancer was believed to always be a fatal disease. Experiences of members of the community were most influential on the formation and maintenance of such beliefs, despite media campaigns and news items promoting advances in treatments and improved cancer survival. Witnessing poor cancer outcomes in the immediate social environment, combined with mistrust of official information sources, may over-ride positive media claims and contribute to the prevalence of fatalistic beliefs in deprived communities [21, 22].

Other findings that were likely to be specific to low socioeconomic groups reflected competing priorities and a lack of resources. Competing priorities such as work commitments and the stresses of day-to-day living took precedence over health concerns, particularly when vague symptoms were dismissed as not serious and getting transport to an appointment was difficult. Priorities such as taking unpaid time off work to consult the doctor were weighed against the perceived urgency or need for an appointment. Once an individual overcame the barriers associated with getting to an appointment, we found evidence of further obstacles to full and effective disclosure of symptom concerns at a service and organisational level. Not being able to communicate symptom concerns effectively in a time limited appointment, and reported policies that preclude discussion of more than one symptom during a consultation, were likely to limit presentation of a cancer symptom. This was especially likely for those who presented with another health complaint to ‘test the water’ before disclosure of a worrying or embarrassing symptom, potentially prolonging disclosure of symptoms [31].

Findings relating to beliefs about cancer translating into either short or long patient interval might be explained by Type I and Type II responses to symptoms [32, 33]. All participants initially disclosed fearful, highly emotive beliefs when asked to think about cancer as a disease (Type I), potentially reflecting a community wide response to cancer. However, after consideration, some participants expressed positive beliefs about the benefits of early diagnosis, which could represent participants using their slower, more deliberate appraisal processes (Type II) [32, 33]. The latter response may prompt symptom presentation, with fear of a late diagnosis of cancer and perceptions of self-efficacy around knowing what to do with a symptom and the ability to discuss concerns, motivating the individual to seek medical help quickly [34]. Using the lay system of healthcare [35–37] to discuss symptoms with family members or friends before visiting the family physician was the norm within the community, to decide whether a medical appointment was necessary. A previous study found that those from a low socioeconomic group were more likely to have a longer patient interval following disclosure of symptoms to a family member or friend [38]. It is likely that if symptom advice is sought from family member or friend within the community who has poor knowledge of the symptoms of cancer and negative beliefs about cancer, poor advice might be given and patient interval lengthened due to false reassurance.

The COM-B model [26] appeared highly applicable in the context of cancer symptom presentation behaviour, allowing exploration of how individual cognitive and affective processes and the wider social context influence behaviour. The model currently represents a bi-directional influence of Capability, Opportunity and Motivation on Behaviour and suggests that both Capability and Opportunity influence Motivation [26]. However, we also found that knowledge of the causes of cancer and symptoms of cancer (Capability) was influenced by the experiences of other people within their social network who have had cancer (Opportunity) [26]. This interaction is not currently represented in the COM-B model, and could be explored in future research into the social determinants of cancer help seeking behaviour.

Limitations of the study included the use of retrospectively recalled or anticipated time to cancer symptom presentation, where biased recall or hypothetical intentions might not reflect actual symptom presentation behaviour, respectively [25]. In addition, framing the study specifically around cancer may have affected study participation in a population of people who are fearful of cancer. Study designs exploring barriers to symptom presentation in a community sample who disclose symptoms, without mention of cancer, could overcome these limitations [39, 40]. The influence of other factors such as age, marital status and gender were not explored in this study, although have been reported to influence the patient interval [9, 37]. Furthermore, in order to understand the barriers to cancer symptom presentation for the age group in which cancer is most common [27], all participants were over the age of 50. Therefore, it is unclear if younger age groups are likely to experience different or additional barriers to cancer symptom presentation behaviour. Finally, this was a UK based study, where access to healthcare provision is free. In countries where patients are required to pay for healthcare consultations and treatment, costs incurred from seeking medical help can influence the decision to seek medical help particularly for those who are income deprived [41–43].

Future interventions targeted at low socioeconomic groups to encourage earlier cancer symptom presentation should take into account the wider influences on symptom presentation behaviour within social networks, for example through a community based educational programme. Such interventions could use social networks to increase community wide knowledge about non-specific cancer symptoms, highlight the significance of multiple symptoms and break down negative beliefs surrounding cancer, reinforcing positive messages about the benefits of early diagnosis and advances in modern treatments. They might also seek to empower people, perhaps through question and topic prompts, to be able to discuss symptoms and concerns with primary care providers.

## Conclusion

Cancer symptom presentation behaviour is influenced by socio-environmental factors. In order to reduce socioeconomic inequalities in cancer outcomes, it is important to understand the wider community and societal influences on behaviour. Interventions, targeted at the specific community, might then be able to encourage timely cancer symptom presentation and earlier diagnosis of cancer among people from low socioeconomic groups.

## Abbreviations

GM: Grace McCutchan
HCP: Healthcare Professional
ICBP: International Cancer Benchmarking Partnership
NAEDI: National Awareness and Early Diagnosis Initiative
UK: United Kingdom
WIMD: Welsh Index of Multiple Deprivation
